# Case Report: A myasthenia gravis patient complicated with renal failure was effectively treated with efgartigimod

**DOI:** 10.3389/fimmu.2025.1526975

**Published:** 2025-03-27

**Authors:** Jia Ke, Qi Zhao, Na Wang, Bei Zhang, Jin-Quan Hu

**Affiliations:** Department of Neurology, Taihe Hospital, Hubei University of Medicine, Shiyan, Hubei, China

**Keywords:** myasthenia gravis, efgartigimod, hemodialysis, chronic kidney disease, myasthenic crisis

## Abstract

Myasthenia gravis (MG) is a neuromuscular junction disorder clinically characterized by fluctuating muscle weakness, in which some patients with respiratory muscle weakness are at risk of progressing to myasthenia gravis crisis and respiratory failure, requiring treatment with rapid antibody clearance. Currently widely used intravenous immunoglobulin and plasma exchange therapy remains ineffective in some patients and is limited by multiple contraindications. Efgartigimod is a newly approved FcRn antagonist for the treatment of myasthenia gravis, which rapidly cleans IgG antibodies in the body, but there is still a lack of guidance on the use of efgartigimod in patients with renal insufficiency. Here, we report a case of MG patient with end-stage renal disease undergoing maintenance hemodialysis who successfully navigated a myasthenic crisis and achieved significant clinical remission through efgartigimod therapy. Moreover, sustained efgartigimod maintenance therapy enabled achievement of clinical minimum state. This case demonstrates the therapeutic potential of efgartigimod in MG patients with concomitant renal impairment and provides clinical evidence supporting its application in this special population.

## 1 Introduction

Myasthenia gravis is a rare neuroimmune disorder characterized clinically by fatigue-related muscle weakness, in some patients by ocular muscle weakness manifested by ptosis and diplopia, in others by trunk muscle weakness such as head ptosis and limb weakness, and in others by respiratory and pharyngeal muscle weakness manifested by dysarthria, dyspnea, and dysphagia (1). Up to 18% of MG patients may experience life-threatening myasthenia crisis and require tracheal intubation for assisted ventilation (2). The pathogenesis of MG is mediated by immunoglobulin G (IgG) antibodies, which include acetylcholine receptors (AchR), muscle-specific tyrosine kinase (MUSK), and lipoprotein receptor protein 4 (LRP4). Among them, AchR antibodies (AchR-Ab) are the most common in MG patients, accounting for about 85% (3). Based on its pathogenesis, the acetylcholinesterase inhibitor pyridostigmine has been recommended as a first-line drug for MG patients and has been suggested to be used in combination with corticosteroid or non-steridal immunosuppressive treatment (4). However, long-term use of corticosteroids has been associated with a range of adverse effects, including diabetes, hypertension and osteoporosis, and the immunosuppressive agents widely suppress the immune system, increasing patients’ risk of infection (4). In addition, approximately 15% of patients continue to experience disease progression despite the aforementioned drug treatment, and even develop drug resistance. Many patients impose a heavy burden on their families and society due to their disabled state and poor quality of life (5, 6). There is an urgent need for more effective, more precisely targeted, and less side-effecting MG treatment drugs.

Efgartigimod is an engineered human IgG fragment. Compared with endogenous IgG, it has stronger affinity for neonatal Fc receptor (FcRn), thereby reducing the recycling of human IgG and increasing the degradation of IgG (7). It is the first FcRn antagonist approved by the United States, Japan, Europe, and China for the treatment of generalized AchR-Ab MG (gMG) (8). International clinical trials and cohort studies in China have demonstrated the clinical efficacy of efgartigimod (9, 10). However, as a new drug, guidance on the clinical application of efgartigimod still needs to be gradually improved. There is no need to adjust the dose in patients with mild renal function injury, but no special pharmacokinetic study has been carried out in patients with renal function injury (11). Reports on the use of efgartigimod in patients with abnormal renal function MG are lacking, and we report here a case of a MG patient with end-stage renal disease undergoing maintenance hemodialysis successfully survived gravis crisis after the use of efgartigimod.

## 2 Case description

A 61-year-old male was first diagnosed with gMG in April 2023, when he presented with left-sided ptosis. The patient’s clinical symptoms included ptosis, diplopia, blurred vision, occasional dysphagia, and weakness of the extremities, with the characteristics of light in the morning and heavy in the evening. The patient had hypertension and was taking amlodipine, metoprolol, and nifedipine to control his blood pressure, and had a history of coronary artery disease, which was treated with aspirin and atorvastatin. He was diagnosed with uremia 10 years ago and received hemodialysis for a long time. At present, the hemodialysis treatment frequency is 5 times/2 weeks.

The physical examination of the patient showed that the left eyelid ptosis, reduced eye fissure, mild limb weakness and difficulty in swallowing solid food. After admission, the patient underwent relevant examinations. Repeated electrical stimulation examination showed that the low-frequency amplitude of the left orbicularis oculi muscle and the left trapezius muscle decreased. The neostigmine test was positive (the ptosis symptoms of the patient were significantly relieved 20 minutes after injection), and the serum anti-AChR IgG (1:32) and anti-titin IgG (1:320) was positive. Chest CT showed no thymoma. The serum antinuclear antibody spectrum, antineutrophil cytoplasmic antibody spectrum, thyroid hormone, tumor marker levels and infectious disease indicators were negative. Blood analysis showed that the patient had mild anemia, increased serum potassium level (5.35 mmol/L), increased creatinine by 711 μmol/L, and no abnormal liver function was found. The patient denied any family history of myasthenia gravis, other autoimmune diseases (e.g., systemic lupus erythematosus, rheumatoid arthritis, and autoimmune thyroid disease), or neuromuscular disorders. Genetic testing (e.g., for congenital myasthenic syndrome-related genes) was not performed, as the patient had no early-onset symptoms (adult onset) or features suggestive of congenital myasthenia (e.g., neonatal manifestations, familial clustering).

Based on the above examination results, the patient was diagnosed with gMG (Myasthenia Gravis Foundation of America [MGFA] IIIa (12), Quantitative MG [QMG] score 11) (13). Therefore, the patient received pyridostigmine treatment at an initial therapeutic dose of 90 mg/day. The ptosis improved after taking the drug, but the patient had obvious abdominal pain, diarrhea, *etc.*, and diarrhea more than 10 times a day in severe cases. Reducing the dose of pyridostigmine could not alleviate the adverse reactions, so pyridostigmine treatment was discontinued, and anti-immunotherapy was added with prednisone 30 mg/day, and a gradual increase in the dosage was carried out. In June 2023, the patient’s condition was stabilized, and prednisone was increased to 40 mg/day, during which he continued to receive regular dialysis treatment. After 5 months, the patient’s condition was stable, and the prednisone began to gradually reduce.

In January 2024, the patient was re-admitted to the Department of Neurology for bilateral lower extremity weakness. The patient had weakness of the extremities, neck muscles, and dysphagia. He was tested with a QMG score of 18 points and MG-specific activities of daily living scale (MG-ADL) score of 9 points (14), and water swallowing test grade 3. Considering that the patient’s symptoms acutely exacerbated, the patient was again treated with the pyridostigmine, and the concomitant application of anisodamine was added to counteract the cholinergic effects of pyridostigmine. In addition, tacrolimus was used for modulating the immune response. After treatment, the patient’s symptoms gradually relieved (QMG 11), and the patient was discharged from the hospital with a treatment regimen of prednisone (25 mg/d), pyridostigmine (90 mg/d), tacrolimus (2 mg/d). In April 2024, the patient once again visited the hospital for bilateral lower extremity weakness, dysphagia, and diplopia. The manual muscle test (MMT) showed that the muscle strength of both upper limbs was grade 4, and the muscle strength of both lower limbs was grade 3, QMG score was 26, MG-ADL score was 12, and water swallowing test grade 3. The patient was admitted with a combination of upper respiratory tract infection, phlebitis with vascular occlusion of the right upper extremity, and acute gastroenteritis, and was admitted with symptoms of vomiting, diarrhea, cough, and sputum.

The patient had a rapid exacerbation of symptoms and a tendency to progress to myasthenia gravis crisis, requiring fast-acting anti-immune therapy. Plasma exchange and intravenous immunoglobulin (IVIg) are recommended treatments for the acute phase of myasthenia gravis (4). However, the patient was comorbid with various comorbidities such as renal failure, infections, and venous thrombosis, and there were contraindications to the above treatments and serious risks of adverse effects. The therapeutic strategy in this case centered on the initiation of efgartigimod, a FcRn inhibitor. The patient was administered once weekly (10 mg/kg) for a total of four weeks and the next cycle of treatment was initiated again at an interval of 1 month. The patient needs to receive hemodialysis treatment 2-3 times a week, in order to avoid hemodialysis affecting the drug absorption, each infusion of efgartigimod was selected after the patient’s dialysis. Currently, the patient has received 3 cycles of efgartigimod.

**Figure 1:**
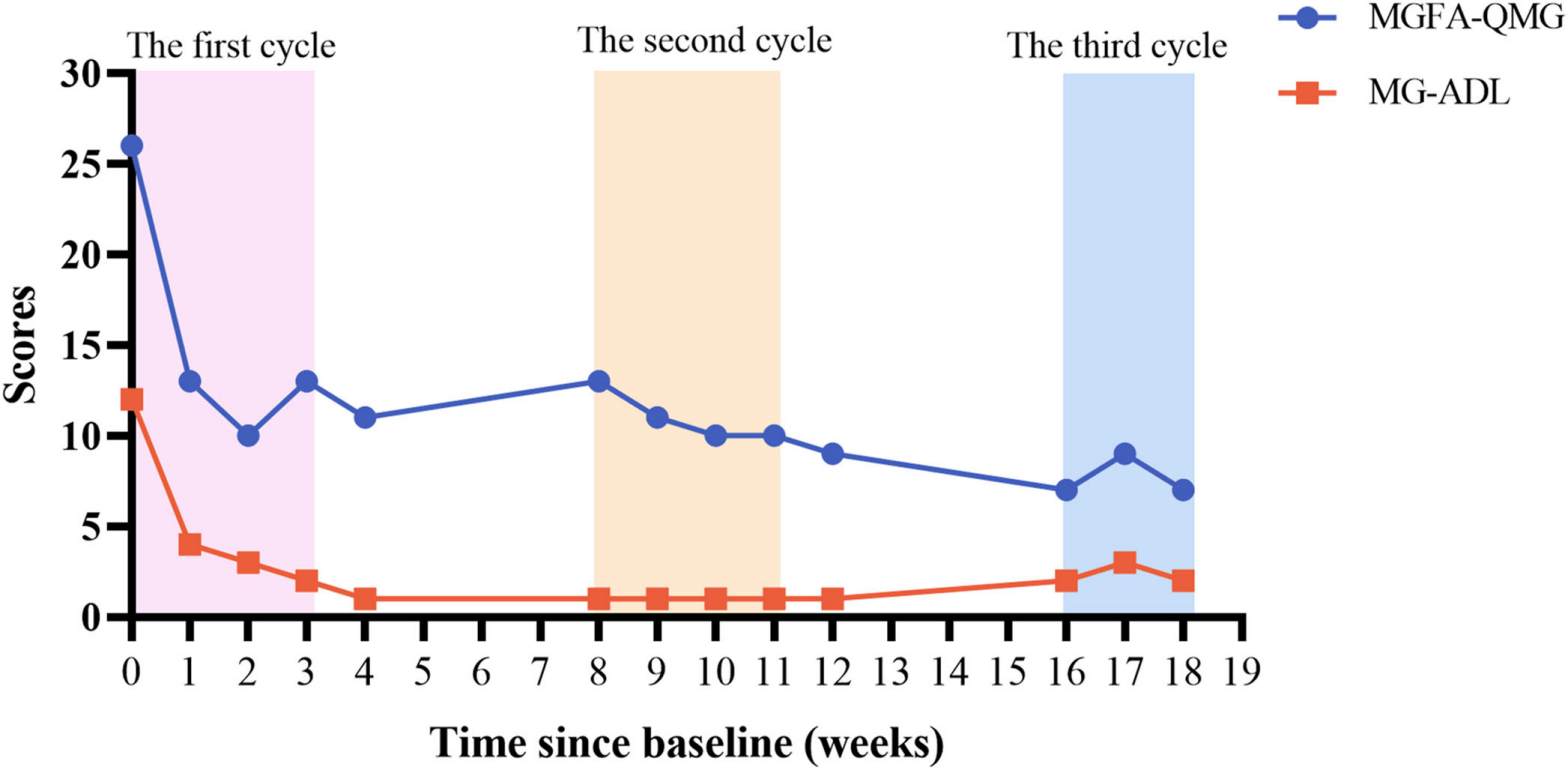
Changes of QMG and MG-ADL scores.

**Figure 2:**
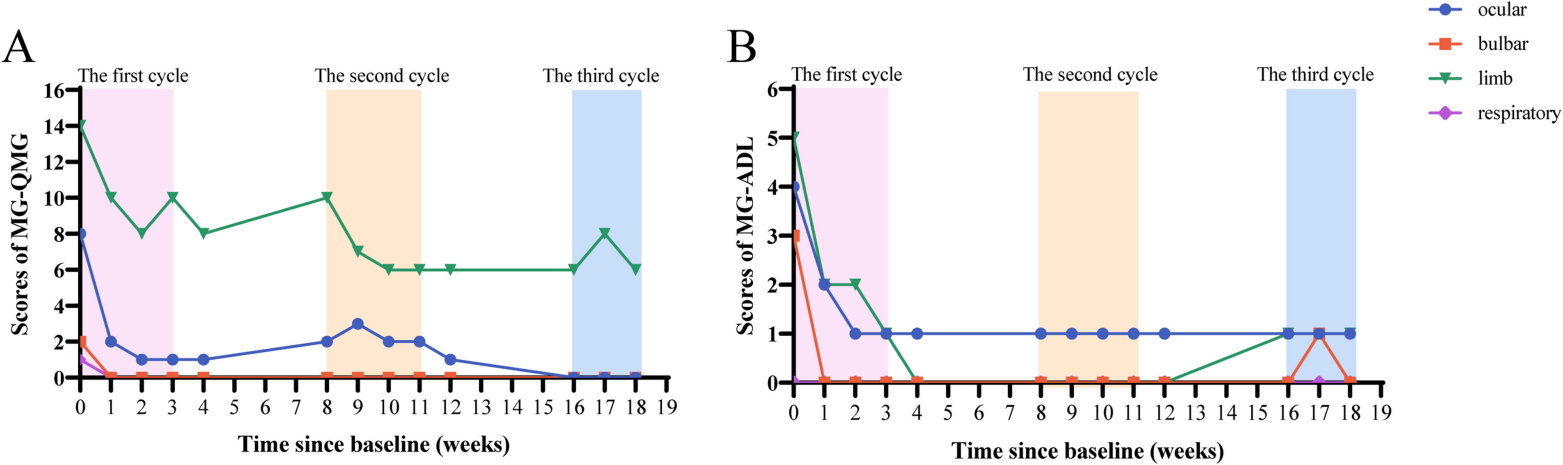
Changes of subdomains of QMG **(A)** and MG-ADL **(B)** scores.

We recorded the QMG and MG-ADL scores of the patient after treatment (**Figure 1**). One week after receiving the first injection of efgartigimod, the patients’ limb weakness and diplopia symptoms rapidly improved. Before the second injection of efgartigimod, the QMG score decreased to 13, and the MG-ADL score decreased to 4, which achieved clinically meaningful improvements (10, 15). In the subgroup classification of the scores (**Figures 2A, B**), weakness symptoms improved in all muscle groups, with the most pronounced decrease in limb and eye muscle. The patient’s scores trended steadily downward throughout the 3-month therapeutic intervention, and there was no significant worsening of symptoms, even during the inter-treatment period. By the time of the most recent treatment with efgartigimod, the patient’s QMG score had decreased to 7, and the MG-ADL score was 2. Minimal manifestations status (MMS) had been achieved (4, 15, 16).

**Figure 3:**
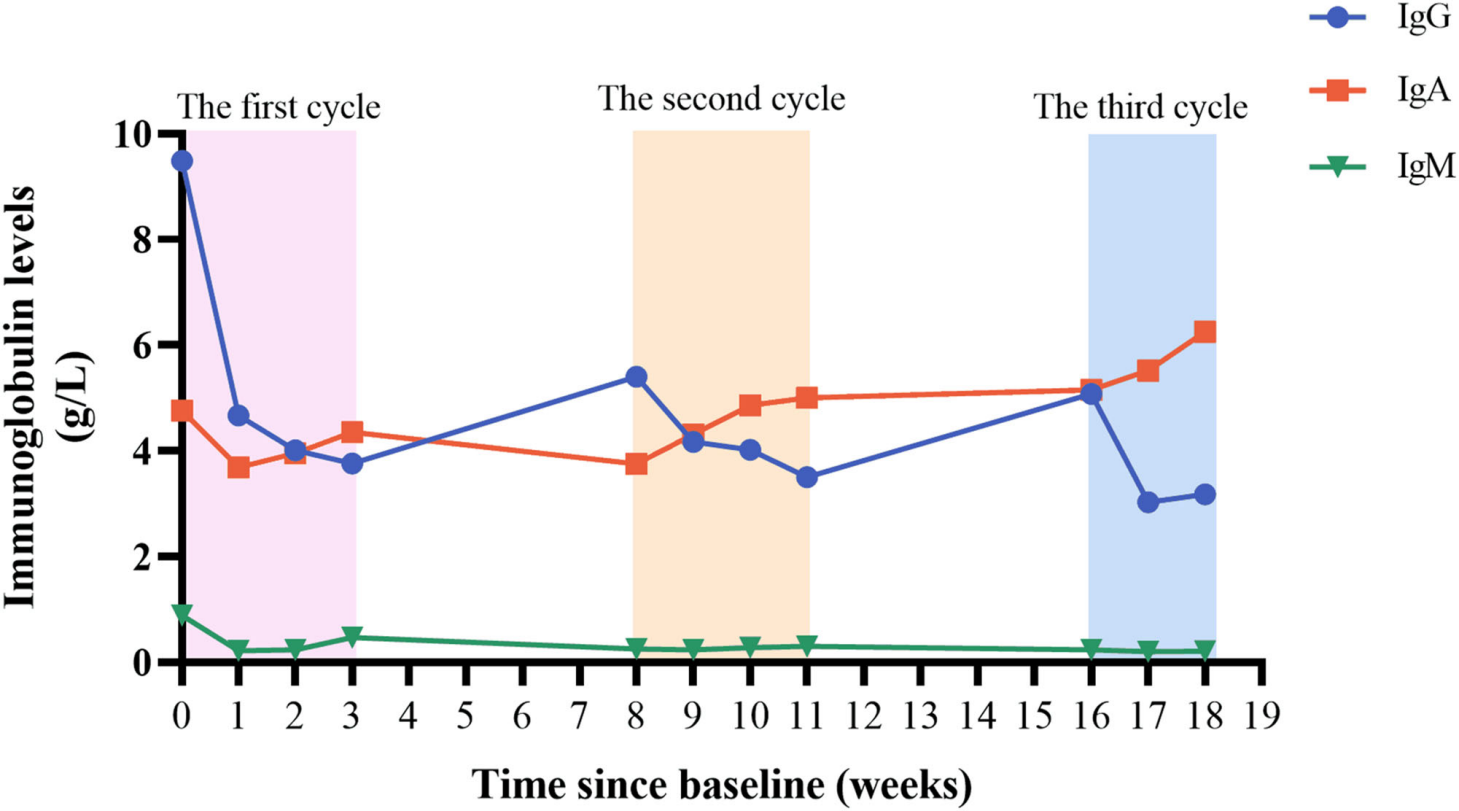
Changes of serum IgG, IgM, and IgA levels.

**Figure 4:**
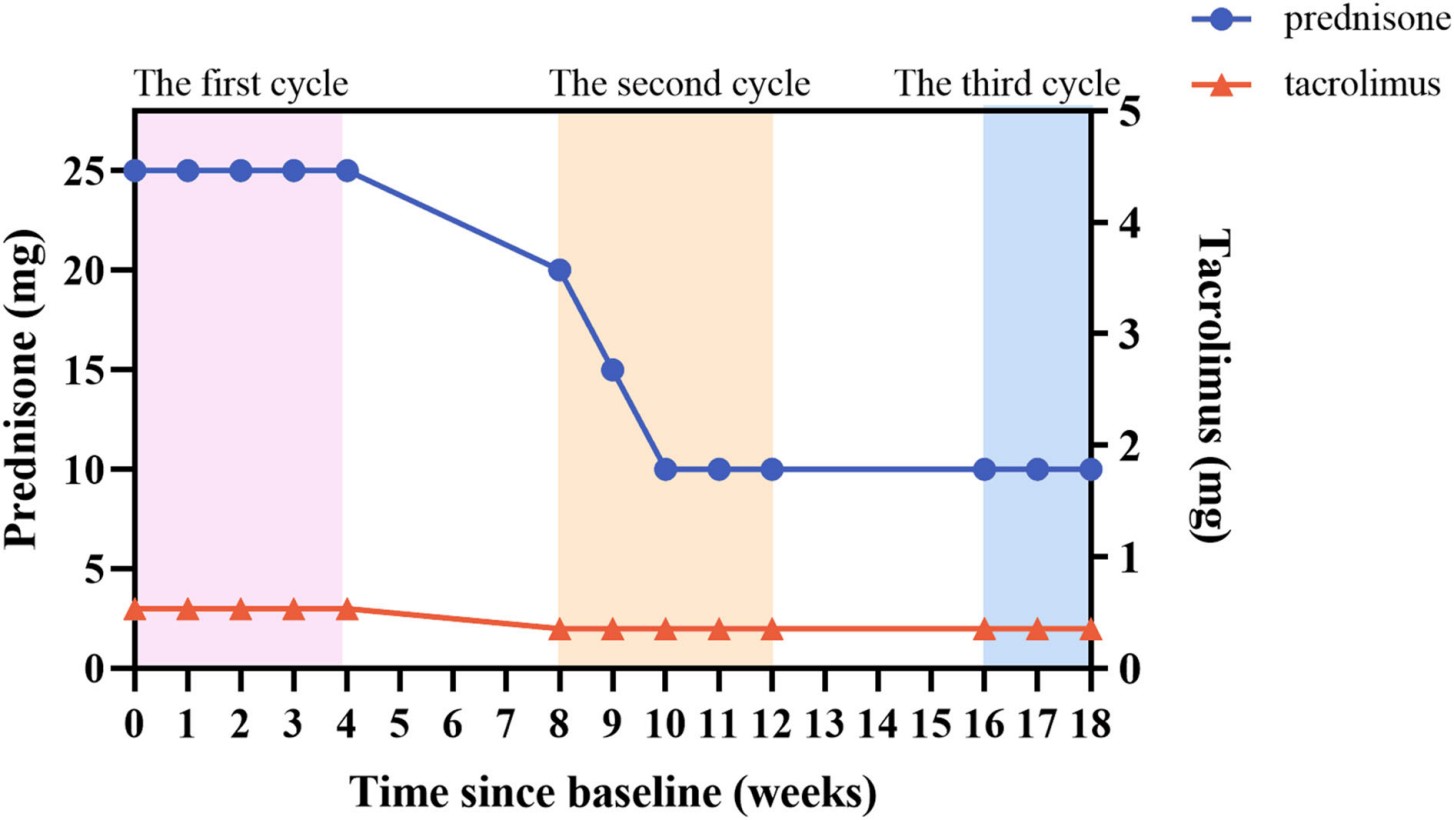
Changes in doses of prednisone and tacrolimus.

During the treatment period, we monitored the patient’s serum immunoglobulin levels (**Figure 3**). In the first week after treatment, the patient’s serum IgG level decreased from 9.49 g/L to 4.67 g/L, which was consistent with the improvement of the patient’s clinical symptoms. There was a slight increase in IgG level between inter-treatment interval, but they were lower than initial levels, suggesting a sustained *in vivo* effect of efgartigimod. The patient’s IgG level had fallen to 3.18 g/L after the most recent treatment, and we suspended the fourth injection of the third cycle because of concerns that the excessively low IgG level increased the patient’s risk of infection. Efgartigimod showed no impact on albumin and other types of globulins. During the patient’s treatment with efgartigimod, the patient continued to be treated with prednisone and tacrolimus, with the dose of the prednisone reduced from 25 mg/d to 10 mg/d, and the dose of tacrolimus had been reduced to 2 mg/d (**Figure 4**). The patient did not experience any symptoms of acute infection, such as fever or cough, throughout the treatment period. In the monitoring of the patient’s blood analysis, liver and kidney function, and serum albumin level, there were no negative effects associated with the efgartigimod treatment.

## 3 Discussion

The development of MG is associated with immune disorders in human that lead to the production of AchR-Ab, which contribute to the pathogenesis of MG through three mechanisms (17): the first mechanism involves the blocking of AchR signaling by AchR-Ab, which impedes neuromuscular signal transduction. The second mechanism involves cross-linking of AchR-Ab to AChR, causing internalization of the AchRs and resulting in a reduction in the number of available receptors. The third mechanism involves activation of the classical complement pathway by AchR-Ab, leading to disruption of the postsynaptic membrane of the motor endplate and a reduction in the number of AChRs. Therefore, in the treatment of rapidly progressive stage of MG, rapid antibody clearance is essential to avoid myasthenic crisis in patients, and traditional therapies are plasma exchange and IVIg (4). However, nearly 30% of MG patients respond poorly to IVIg because of the VNTR polymorphism, and in addition, the above treatments are exacerbated in patients with renal failure, infections, and thrombosis (18–21). In recent years, biological targeted therapy has made great progress in neuroimmune diseases, and is recommended for the treatment of MG. FcRn is a multifunctional FC-γ receptor. FcRn binds to circulating IgG and releases the IgG into the extracellular space, preventing it from being degraded by cytosolic lysosomes. As a result, the half-life of circulating IgG is several times longer than that of IgA and IgM that are not circulated by FcRn (8). The rate of FcRn-mediated IgG recirculation is 40% higher than the rate of IgG production, and IgG recirculation, rather than production, is central to IgG homeostasis (22). Thus, inhibition of FcRn increases IgG catabolism, which provides a new therapeutic opportunity for IgG-mediated autoimmune diseases (7).

The FcRn antagonist efgartigimod, a humanized IgG1 Fc fragment designed by ABDEG (AntiBody that enhances IgG DEGradation) technology, has a stronger affinity for FcRn at neutral and acidic pH. It can inhibit IgG recirculation and increases its catabolism but has no significant effect on other immunoglobulin and albumin levels (8). The rapid efficacy and safety of efgartigimod in MG has been demonstrated in phase 2 and phase 3 clinical trials (NCT02965573, NCT03669588) (10, 23). Based on the encouraging results of the ADAPT study, efgartigimod was approved in the U.S. in December 2021, as well as in Japan and Europe in 2022, and in China in 2023 (8). Numerous studies have demonstrated the promising therapeutic effects of efgartigimod in myasthenia gravis (9, 24).

Efgartigimod has a rapid onset of action with antibody clearance of up to 40% after the first week of administration, is more efficient at clearing IgG than IVIg, and is recommended for the acute phase of MG and for long-term management (10, 11). This is because efgartigimod is a selective IgG antagonist, rather than a large amount of IgG supplement like IVIg, which saturates the FcRn receptor (11). Several studies have reported the effective effects of efgartigimod in the acute phase of MG, but there are no reports on its use in patients with severe renal impairment (25–27). No specific pharmacokinetic studies of efgartigimod have been performed in patients with renal or hepatic impairment; however, population pharmacokinetic analyses based on data from clinical studies have shown that, compared with healthy volunteers with normal renal function, human with mild renal impairment have a 22% increased exposure to efgartigimod and that efgartigimod use did not induce renal impairment in human (8, 10, 11).

In this case report, the patient had renal failure and needed hemodialysis 2-3 times per week, and at the same time, the patient had contraindications to plasma exchange and IVIg due to infections and thrombosis, and had a severe diarrheal reaction to pyridostigmine, which is a refractory form of MG (28). The treatment with efgartigimod still showed a surprisingly effective effect, and there were no adverse reactions, which indicated that the rapid and effective effect of efgartigimod on MG, with little nephrotoxicity, is safe to use in cases of comorbidities such as thrombosis and infections, and therefore has narrower restrictions on its use.

After the first medication, the symptoms of this patient improved rapidly and achieved clinically significant improvement. This change was consistent with the decrease of serum IgG level, indicating that efgartigimod acted rapidly *in vivo*. Thereafter, the patient followed the dosing regimen in the ADAPT study, with weekly dosing in the first cycle and the next cycle starting after a 1-month interval. Even in the inter-treatment period, the patient’s symptoms did not show significant aggravation, and the serum IgG level did not rebound significantly, suggesting the long-lasting effect of efgartigimod. At the same time, the patient’s prednisone dose was gradually reduced during the use of efgartigimod, and the complete discontinuation of pyridostigmine still controlled the myasthenia symptoms, which suggests that efgartigimod may be able to replace the traditional first-line drug of MG.

The reasons for the rapid effect of efgartigimod on are as follows: first, efgartigimod can be rapidly distributed in the body and sustained action. Plasma drug concentration peaks 2 hours after the intravenous infusion of efgartigimod and it can be efficiently distributed from the circulation to the tissues (11). Secondly, efgartigimod has a high IgG clearance rate. In our patient, serum IgG decreased by approximately 50% after one week of dosing. Studies have shown that human serum IgG begins to decline as early as 1 day after the administration of efgartigimod, and the decline reaches its maximum on the sixth day after administration, with a maximum decrease in IgG levels of 70% during the first cycle of administration (11, 23). It has also been observed in Fcgrt^-/-^ mice that the half-life of IgG is reduced from 6-8 days to about 1 day (29). In addition, efgartigimod has a persistently effects *in vivo*. It has been reported that efgartigimod remains measurable in patient 21 days after the last dose, and that the IgG level-lowering effect of efgartigimod persists up to the 29^th^ day after the first infusion (11, 23). In the ADAPT study, 1/3 of the patients maintained clinically meaningful improvement for more than 12 weeks (10). This is related to the fact that efgartigimod can increase the body’s postsynaptic membrane AchR reserve, restoring and maintain neurotransmission (10, 30). Also, studies have shown that efgartigimod can be recycled from cells, increasing the duration of action without causing efgartigimod accumulation, which accounts for the drug’s long-lasting effects (11).

In this case, the patient was required to undergo hemodialysis 2-3 times per week, and this is the first report on the application of efgartigimod in MG patients with chronic kidney disease. Efgartigimod is decomposed into small peptides and amino acids in the body, and finally excreted in the urine or reused (11). The molecular weight of efgartigimod is 53kDa. During hemodialysis, only molecules with a relative molecular weight of less than 500 Da pass through, that is, only small molecules can be removed from the blood, while the clearance rate of medium and large molecules is very low (31). This suggests that hemodialysis will not affect the concentration of efgartigimod in the body. Moreover, in our follow-up, the patients did not experience any deterioration in renal function due to the use of efgartigimod, suggesting that efgartigimod does not cause significant renal damage, but more pharmacokinetic studies of efgartigimod in patients undergoing hemodialysis treatment should be conducted in the future to better guide its clinical application.

In addition, the patient had complications such as infection and thrombosis before medication, and the use of efgartigimod did not aggravate the above situation, which was also not reported in previous cases of efgartigimod application. No infection occurred during the use of efgartigimod in this patient. This is because efgartigimod selectively reduces IgG without affecting the production or quality of IgG and did not affect other components of the innate or adaptive immune system (11, 32). This is consistent with our follow-up results: efgartigimod had no significant effect on the levels of IgA and IgM.

The dosing regimen adopted by this patient was consistent with that of the ADAPT study, with weekly infusions for 4 weeks, a 1-month cessation before starting the next cycle of treatment. After the two fixed treatment cycles reached the MSE, the time of the next treatment cycle was determined according to the clinical evaluation results of the patient (10). At present, there is no guidance on whether MG patients need long-term treatment with efgartigimod. After 2 cycles of treatment, this patient continued the third cycle. During the treatment period, the patient’s symptoms did not fluctuate significantly, but after the third treatment of the third cycle, the patient’s IgG level was too low, so efgartigimod treatment was suspended. Therefore, more studies are needed to guide the long-term treatment clinic of efgartigimod. A recent study showed that using a continuous dosing regimen (every 2 weeks) after the first cycle of treatment had similar clinical effects and antibody clearance as the fixed-cycle regimen, but the continuous dosing regimen had less fluctuation in MG-ADL and smoother symptoms (33), which provides more options for the clinical application of efgartigimod. Subcutaneous formulations of efgartigimod are available and studies have shown that subcutaneous administration has similar effects to intravenous administration, which will lead to more convenient dosing and less restrictive dosing for patients in the future (34).

In this case report, the QMG and MG-ADL scores were employed to evaluate symptom improvement in patients. This result meets the strict mitigation criteria: MG-ADL ≤2, QMG ≤7 (35). Notably, a divergence between QMG and MG-ADL scores emerged during the 2nd cycle (elevated QMG score juxtaposed with reduced MG-ADL score). This phenomenon represents a natural consequence of MG heterogeneity and scales design differences rather than data inaccuracies. The QMG and MG-ADL scales capture distinct clinical dimensions: the former quantifies objective neuromuscular impairment through physical examinations, while the latter assesses subjective functional limitations in daily living. Cutoff values for strict MM-or-better were MG-ADL ≤2, QMG ≤7 (sensitivity 82.0% and 88.7%; specificity 85.0% and 70.0%; and accuracy 91.2% and 88.7%, respectively) (35). The observed 2nd cycle divergence may be due to the fact that patient maintained function through behavioral adaptation despite the persistence of objective weakness. Subsequent convergence in the 3rd cycle likely indicates true clinical remission. The QMG scale includes many limb strength measurements, which may not align with subjective perceptions in patients engaged predominantly in low-demand daily activities. In future studies, it’s very necessary for multidimensional assessment protocols incorporating complementary metrics (QMG, MG-ADL, and MGC [Myasthenia Gravis Composite]) to enhance measurement accuracy. This case reveals the asynchrony of objective and subjective improvement during the course of treatment, which has clinical warning values. Clinically, the objective strength deterioration (detected by QMG) may precede functional decline, so QMG should be closely monitored in the clinic to prevent myasthenic crises.

## 4 Conclusion

In conclusion, our case report suggests that efgartigimod acts rapidly in patients with acutely exacerbated gMG to alleviate the symptoms, and can be used as a therapeutic drug in the acute and maintenance phases of myasthenia gravis. Moreover, hemodialysis treatment has no significant effect on the efficacy of efgartigimod and does not aggravate renal impairment in patients. This is the first case of the application of efgartigimod in MG patients who are also undergoing hemodialysis treatment, which provides clinical experience for the applications in other patients combined renal end-stage. In the future, the pharmacokinetic study of efgartigimod should be carried out in patients with renal function impairment *in vivo*, which can provide more guidance for the application. Meanwhile, our case also suggests that efgartigimod is a promising drug choice for MG patients with refractory myasthenia gravis and complex disease.

## Data Availability

The raw data supporting the conclusions of this article will be made available by the authors, without undue reservation.
